# Osteocytic Pericellular Matrix (PCM): Accelerated Degradation under In Vivo Loading and Unloading Conditions Using a Novel Imaging Approach

**DOI:** 10.3390/genes13010072

**Published:** 2021-12-28

**Authors:** Shaopeng Pei, Shubo Wang, Jerahme R. Martinez, Ashutosh Parajuli, Catherine B. Kirn-Safran, Mary C. Farach-Carson, X. Lucas Lu, Liyun Wang

**Affiliations:** 1 Center for Biomechanical Engineering Research, Department of Mechanical Engineering, University of Delaware, Newark, DE 19716, USA; shaopeng@udel.edu (S.P.); shubow@udel.edu (S.W.); Jerahme@udel.edu (J.R.M.); parajuli@udel.edu (A.P.); lywang@udel.edu (L.W.); 2 Department of Biological Sciences, University of Delaware, Newark, DE 19716, USA; cbsafran@widener.edu; 3 Department of Biology, Widener University, Chester, PA 19013, USA; 4 Department of Diagnostic and Biomedical Sciences, School of Dentistry, University of Texas Health Science Center, Houston, TX 77054, USA

**Keywords:** osteocyte, click-chemistry, metabolic labeling, tibia loading, hindlimb suspension, pulse-chase experiment

## Abstract

The proteoglycan-containing pericellular matrix (PCM) controls both the biophysical and biochemical microenvironment of osteocytes, which are the most abundant cells embedded and dispersed in bones. As a molecular sieve, osteocytic PCMs not only regulate mass transport to and from osteocytes but also act as sensors of external mechanical environments. The turnover of osteocytic PCM remains largely unknown due to technical challenges. Here, we report a novel imaging technique based on metabolic labeling and “click-chemistry,” which labels de novo PCM as “halos” surrounding osteocytes in vitro and in vivo. We then tested the method and showed different labeling patterns in young vs. old bones. Further “pulse-chase” experiments revealed dramatic difference in the “half-life” of PCM of cultured osteocytes (~70 h) and that of osteocytes in vivo (~75 d). When mice were subjected to either 3-week hindlimb unloading or 7-week tibial loading (5.1 N, 4 Hz, 3 d/week), PCM half-life was shortened (~20 d) and degradation accelerated. Matrix metallopeptidase MMP-14 was elevated in mechanically loaded osteocytes, which may contribute to PCM degradation. This study provides a detailed procedure that enables semi-quantitative study of the osteocytic PCM remodeling in vivo and in vitro.

## 1. Introduction

Pericellular matrix (PCM), a thin proteoglycan-rich cellular coating, is found around nearly all types of cells, including yeast [1], endothelial cells [2], epithelial cells [3], fibroblasts [4], smooth muscle cells [4], chondrocytes [5], and osteocytes [6,7]. As a molecular sieve, PCM regulates the transport of nutrients, waste, and signaling molecules (growth factors, cytokines, and hormones) to and from living cells [2,5,8]. PCM components such as perlecan also can bind/sequester various ligands and serve as co-receptors to plasma membrane receptors [2,5,9]. In bones, PCM plays a critical role in the survival and function of osteocytes [6,8,9], which are the primary mechanical sensing cells [10,11] and the master orchestrators of osteoblasts and osteoclasts during bone adaptation to mechanical cues [12,13]. Osteocytic PCM fills the extracellular space (0.1–1 μm) surrounding the cell [6,14], interacts with fluid flow driven by external loading [10,15,16], and results in a cascade of mechanobiology-driven processes [12,13,17,18]. The cascade begins with acute responses of osteocytes—elevated intracellular calcium [19,20] and release of secondary messengers (e.g., prostaglandin E2, adenosine triphosphate, and nitric oxide) [12,13,17]. Secondary signal activation associated with ECM-receptor interaction results in reorganization of the cytoskeleton [21] and regulation of intracellular pathways such as the Wnt/β-catenin pathway [13,18,20,22]. These responses eventually converge to osteoblastic bone formation and/or osteoclastic resorption, which are modulated by osteocyte-secreted sclerostin [23] and receptor activators of nuclear factor kappa-Β ligand (RANKL) [24], respectively. These signaling molecules need to pass through the osteocytic PCM within the bone lacunar-canalicular system (LCS) before reaching their target cells [8]. Thus, the structure, composition, and mechanical properties of osteocytic PCM influence both mechanosensing and cell–cell signaling in bone remodeling.

In contrast to the sparser osteocytic PCM documented in mice with perlecan/HSPG2 deficiency and aging [25], individual PCM tethers—functioning as molecular sensing apparatus [15,25]—are typically thought to be very stable, detecting mechanical signals repetitively and consistently. Considered in this manner, the integrity of the PCM tether structure is itself essential to the maintenance of normal mechanosensing. However, PCM undergoes continuous turnover in other mechanosensitive cells such as endothelial cells [2] and chondrocytes [26]. Endothelial cells shed PCM glycocalyx components under continuous blood flow; the shedding/degradation of glycocalyx can be exacerbated by elevated oxidative stress, inflammatory cytokines [27], and high plasma glucose/salt content [28,29]. Rapid turnover of the chondrocyte PCM after joint injury is also considered a hallmark of osteoarthritis [26]. Given these findings, it is reasonable to believe that the osteocytic PCM would turnover as a consequence of the mechanical environment, and PCM synthesis may change with age as observed in other matrices [30].

In order to test these ideas, we developed a new method based on metabolic labeling and bio-orthogonal “click chemistry” [31,32,33], which allowed us, for the first time, to study de novo osteocytic PCM in living bone and cultured cells. The method addressed the technical challenges of measuring osteocytic PCM—small scale and encasement in mineralized bone—and offered advantages over conventional methods. Immunohistochemistry using specific antibodies [7] and staining using glycan-binding lectins [34] work well on fixed samples, but they are unsuitable for in vivo tracking. Radiolabeling with ^35^S allows in vivo tracking [35] but lacks spatial resolution. In this study, the novel labeling method was tested and validated in vitro and in vivo and then applied in classic “pulse-chase” experiments [36] to compare the half-life of the osteocytic PCM in vitro and in vivo as a demonstration of its potential in studying osteocyte mechanobiology. 

## 2. Materials and Methods

### 2.1. Metabolic Labeling of the Osteocytic PCM In Vitro and In Vivo

**Figure 1:**
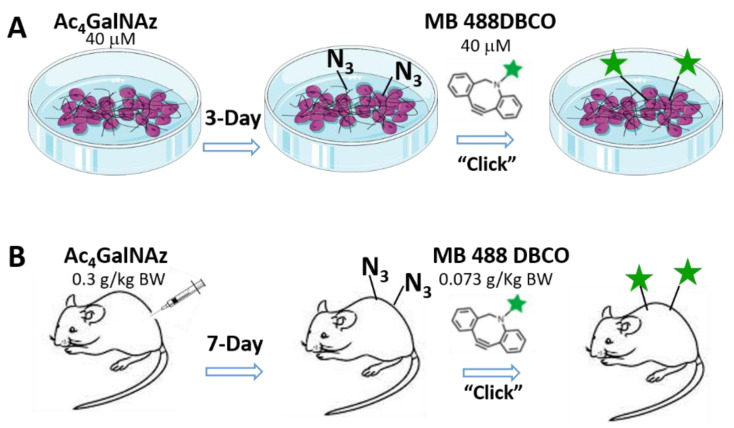
Metabolic labeling of de novo PCM in vitro (**A**) and in vivo (**B**). Cells or animals receive modified sugar molecule (Ac_4_GalNAz) with azide (N_3_) tags, which can be incorporated during the synthesis of new glycans. The newly synthesized glycans bearing the azide groups can click-react biorthogonally with fluorescent tracers carrying the alkyne group (MB 488 DBCO).

Published protocols [32,33] were slightly modified for our in vitro and in vivo experiments (Figure 1). Briefly, an azido analog of GalNAc—Ac_4_GalNAz (azidoacetylgalactosamine-tetraacylated)—was supplemented to osteocytes or injected into mice. GalNAc sugar is a building block for proteoglycans, glycoproteins, and glycosphingolipids [37]. The modified sugar molecules can compete with their prototypes and be metabolized by cells for the synthesis of large glycoconjugates *via* the mucin-type *O*-glycosylation pathway [31]. Thus, newly synthesized cell surface glycans are labeled with azide groups, on which fluorescent tracers carrying alkyne groups can easily react because of the bi-orthogonally “click” chemistry [32,33].

For in vitro experiments (Figure 1A), MLO-Y4 cells were cultured in α-MEM (ThermoFisher Scientific, Waltham, MA, USA) supplemented with 5% (*v*/*v*) FBS/CS and 1% P/S (Hyclone Laboratories Inc., Logan, UT, USA) to reach 60–70% confluency in crystal imaging chambers. The cells were fed with the above culture media (300 μL) containing Ac_4_GalNAz (Click Chemistry Tools, Scottsdale, AZ, USA) at a concentration of 40 μM for three consecutive days. Afterwards, the cells were submerged in 300 μL culture media containing 40 μM fluorescent dye MB 488 DBCO (Click Chemistry Tools) for 0.5 h, during which the “click” reactions could occur. After a thorough wash of MB 488 medium, the cells were dyed with 5 μM CellTracker^TM^ Red (ThermoFisher Scientific) for 15 min to label cell bodies as contrast. Labeled cells were then imaged using a confocal microscope with a 20×/0.5 NA objective (LSM510, Carl Zeiss Inc., Thornwood, NY, USA). We used 488 and 561 nm laser excitations for green and red imaging channels. In order to reduce potential toxicity and side effects from laser exposure, single images were taken under a pinhole of 1 Airy unit and an acquisition time less than 1 s. Experiments were repeated three times.

For in vivo experiments (Figure 1B), 10-week old male mT/mG transgenic mice (stock # 007676, the Jackson Laboratory, Bar Harbor, ME, USA) received intraperitoneally (I.P.) injections of Ac_4_GalNAz dissolved in 20% *v*/*v* DMSO (concentration: 93 mM and dose: 0.3 g/kg body weight) or vehicle control (20% *v*/*v* DMSO) for seven consecutive days, followed by tail injection of MB 488 DBCO dissolved in 20% *v*/*v* DMSO (concentration: 14 mM and dose: 0.07 g/kg BW) on day 8. After a one-day washout period, mice were sacrificed for the harvest of calvaria and tibiae. After soft tissue removal, the bones were immersed in PBS and imaged using a water immersion objective 1 h post sacrifice. In these bones, the constitutive membrane-bound tdTomato signals allowed us to contrast red cell bodies with their surrounding green PCM labels, if any, similarly as did CellTrackerTM Red for cultured cells. Osteocytes 20–30 μm below the flat periosteum of calvaria or the tibial anterior-medial surface were imaged using the confocal microscope (LSM510) with a 63×/1.4 NA objective under a pinhole of 1 Airy unit with the settings (field of view of 512 pixels × 512 pixels, pixel size of 0.39 μm). We have successfully performed such in situ osteocyte imaging using the same microscope setup [25]. Excitations (488 and 561 nm) were used to obtain both red and green signals.

All animal protocols were approved by the Institutional Animal Care and Use Committee (IACUC) at the University of Delaware. The animals were bred and housed in a fully accredited animal facility, and standard animal care regarding temperature, humidity, and light/dark cycle was followed, with free access to normal chow and water.

### 2.2. Testing the Metabolic Labeling Method in Young vs. Old Bone

In order to investigate if our labeling method was sensitive enough to detect PCM changes associated with the aging process, C57BL6/J male mice (the Jackson Laboratory) of 15 or 65 weeks of age (one mouse per age) were metabolically labeled, as described above (Figure 1B). Animals were sacrificed 1 day post MB 488 DBCO injection and both tibiae were dissected and immersed in 70% *v*/*v* ethanol for 1 d, 30% *w*/*v* sucrose for 12 h before being frozen in an optical cutting temperature compound (Tissue-Tek^®^ O.C.T., Sakura Finetek, Torrance, CA, USA). Longitudinal sagittal sections of tibiae (~20 μm thick) were collected without decalcification by using cryosection films (Section-lab, Hiroshima, Japan), as described previously [38]. Ten sections per animal were prepared for quantification. Sections were counterstained with Hoechst 33342 for nuclei visualization and imaged using the LSM510 confocal microscope with a 63×/1.4 NA objective under a pinhole of 1 Airy unit. Z-stack images of tibial cortex (field of view of 512 pixels × 512 pixels, pixel size of 0.39 μm) with a step of 0.32 μm, 90 slices per stack, and one stack per section were used. Using a custom MATLAB program, individual osteocytes within the cortex could be identified by using nuclear staining, and the de novo PCM amount was quantified using average fluorescence intensity over a shell of 1.2 μm surrounding the cell surface, given the gap between the osteocyte and lacunar wall [14]. Only osteocytes with entire cell bodies captured in the 3D stacks (484 and 215 cells from young and old bones, respectively) were analyzed to generate histograms of osteocytic PCM intensity. By pooling all the PCM data, osteocytes were classified into three categories based on PCM intensity: low (those below the mean value minus one standard derivation), high (those higher than the mean value plus one standard derivation), and medium (those in between).

### 2.3. Pulse-Chase Experiments in Mice and Cultured Osteocytic Cells

We further demonstrated an imaging approach in quantifying the half-life of the osteocytic PCM in vivo and in vitro. MLO-Y4 cells were cultured in 24-well plates (at a seeding density of 132 k cells/cm^2^) until approximately 80% of confluency (2–3 d) and then metabolically labeled as described above. Cells were fed with culture media without Ac_4_GalNAz as the vehicle control group. Cells in both groups were exposed to MB 488 DBCO (“pulse labeling”) as described in Section 2.1. In order to avoid photobleaching under laser scanning microscopy, the fluorescence intensity of the de novo PCM within the attached cell culture monolayer was longitudinally measured with a plate reader (SpectraMax^®^ 340PC, Molecular Devices, San Jose, CA, USA) at several time points for up to 5 days. The experiment was repeated five times.

For in vivo pulse-chase experiments, 16 male mT/mG transgenic mice (10-week old) were metabolically labeled with either Ac_4_GalNAz (Figure 1B, 14 mice) or vehicle (20% *v*/*v* DMSO, 2 mice). All mice, including the vehicle controls, were pulse labeled with MB 488 DBCO on day 8. Vehicle control mice were sacrificed one day later to confirm that unbound fluorescent tracers were cleared out of the bone tissue. During the chase phase of experiments, which lasted from 3 to 7 weeks, mice were under mechanical loading (N = 6 mice), hindlimb suspension (N = 5 mice), or ground control (N = 5 mice). All loading or disuse protocols were initiated after the period of sugar injection and pulse labeling. For the mechanical loading group, mice were anesthetized with 3% (*v*/*v*) isoflurane and subjected to axial cyclic compression on the left tibiae at 5.1 N, 4 Hz, 5 min/d, and 3 d/week, similarly to our previous study [25], while the right tibiae served as contralateral non-loaded controls. Due to the long loading duration, the load magnitude was reduced from 8.5 N to 5.1 N in order to avoid potential injury on the knee joint. Please note that the induced surface strain (~680 με) under the current loading regimen mimicked strains experienced by the bone during moderate physical activities. Mice were sacrificed 1 week, 4, or 7 weeks after pulsation (two mice at each time point). For the hindlimb suspension group, mice were tail suspended at approximately 30 degrees to the ground [20]. The tails were wrapped with medical tapes, which then were attached to an overhead bar by using a rotating anchor, allowing radial motions. No surgery was involved. Two mice were suspended separately in a rat cage with a divider. The mice were sacrificed at 1 week (one mouse), 2 weeks (two mice), or 3 weeks (two mice) post pulsing. The duration of hindlimb suspension was limited to three weeks to avoid excessive stress to the animals. Control mice were raised in cages with normal locomotion and sacrificed at 1 d (one mouse), 4 weeks (two mice), or 7 weeks (two mice) after the pulse. Both tibiae were dissected with soft tissues removed and stored in 70% *v*/*v* ethanol. In order to ensure consistent comparisons among multiple groups and time points, plastic-embedded bones and cross sections at specified locations were used. Samples were dehydrated further and embedded in methyl methacrylate [39]. Cross sections located at 30–40% distal from the tibia proximal end, a site experiencing maximal tension and where in situ calcium signaling of osteocytes was imaged [20], were cut and polished to 50 μm of thickness. The entire cross-sectional image of the tibial cortex was obtained using tile imaging with a Zeiss LSM510 microscope equipped with a 20×/0.5 NA objective. Although PCM labeling is blurred during plastic-embedding, areal averaged fluorescence intensity, indicating the amount PCM signals, was measured for the tibial cross section (periosteum and endosteum excluded) using custom MATLAB codes, and two to three slices per bone were analyzed. Linear regression of the cortex labeling intensity vs. time was performed using the Origin^®^ software (OriginLab Corp., Northampton, MA, USA). The half-life for newly synthesized PCM was defined as the time it takes for the PCM intensity to drop to 50% of the initial level minus the background value in the vehicle group.

### 2.4. RNA-Sequencing and Immunohistochemistry

In order to explain the observations of PCM degradation, we analyzed transcripts encoding proteins or enzymes involved in matrix turnover using our RNA-sequencing dataset [20]. In brief, young adult C57BL/6J male mice (N = 6 mice) were loaded for one day or seven days on the left tibia, while the right tibia served as contralateral control. The extracted mRNA samples were sequenced, and differentially expressed transcripts were identified between loaded and non-loaded groups [20]. The loading-induced fold changes for transcripts encoding MMPs, ADAMs, and ADAMTSs and those encoding collagens and proteoglycans were analyzed and shown in a heatmap. Immunohistochemistry (IHC) was performed to reveal the protein expression patterns of specific MMPs. C57BL/6J mice (16 week-old, N = 3 mice) were loaded with 5.1 N peak force for one week on the left tibia, as described above. Both tibiae were harvested, fixed in 4% (*w*/*v*) paraformaldehyde (PFA), decalcified in 10% (*w*/*v*) ethylenediaminetetraacetic acid (EDTA, pH = 7.4) for three weeks, and embedded in paraffin. Sagittal sections (5 μm thick) underwent antigen retrieval with sodium citrate at 60 °C for 12 h, followed by IHC staining of MMP2, MMP9, and MMP14 by using the rabbit IgG primary antibodies from Invitrogen^®^ (Thermo Fisher Scientific) at recommended dilutions (MMP2, 1:500; MMP9, 1:500; MMP14, 1:200) and horse anti-rabbit secondary antibodies (ImmPRESS Horse Anti-Rabbit Polymer kit, Vector Laboratories, Burlingame, CA, USA). Three sections per bone of the diaphysis, located ~30% to the proximal end, were imaged with the 20×/0.5 NA objective from an upright epifluorescent microscope (Axio Imager.A2, Carl Zeiss Inc.). Three views per bone were imaged, with ~10 cells per view. The percentage of positively stained cells over the total number of cells was calculated.

## 3. Results

### 3.1. Metabolic Labeling of De Novo Osteocytic PCM in Cultured Cells and Living Bone

**Figure 2:**
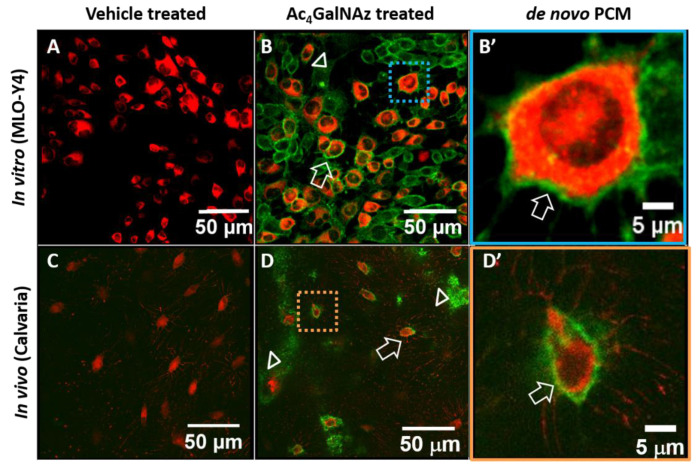
Metabolic labeling of de novo osteocytic PCM in vitro (**A**,**B**,**B’**) and in vivo (**C**,**D**,**D’**). While the vehicle treated cells (**A**) and animal tissues (**C**) showed negligible green signal, green “halos” (arrows) were observed around MLO-Y4 cells (stained in CellTracker^TM^ Red, **B**) and osteocytes in calvaria of mT/mG mice (red, **D**). Green signals (arrow heads) also were observed at regions close to the bone surfaces (**D**). The labeled PCM (arrows) was clearly observed next to the plasma membrane, as shown in magnified views (**B’**,**D’**).

Metabolic labeling of the osteocytic PCM was achieved both in vitro and in vivo (Figure 2). MLO-Y4 cells receiving Ac_4_GalNAz showed a clear and strong green “halo,” while cells in the vehicle control group (DMSO) showed only red stained cytoplasm (Figure 2A,B). Similarly, a negligible green signal was found around osteocytes in the vehicle treated mice (with red tdTomato expression, Figure 2C), while halo-like fluorescent signals were found around osteocytes in mice receiving Ac_4_GalNAz (Figure 2D). In the magnified pictures, de novo PCM was labeled immediately next to the cell bodies (arrows, Figure 2B’,D’).

### 3.2. Different Labeling Patterns in Young vs. Old Bone

**Figure 3:**
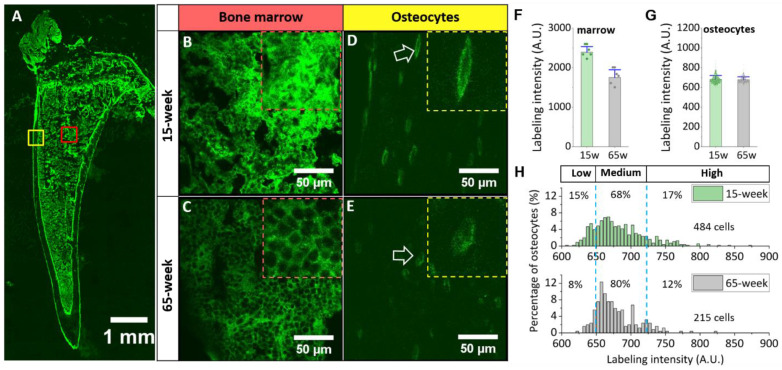
Different PCM labeling patterns in young vs. old bone. (**A**) A sagittal tibial section showed stronger green signals at the periosteum, endosteum, primary spongiosa, and marrow (red box) than in cortical bone (yellow box). (**B**,**C**) In marrow bone, stronger signals were observed in young (**B**) than old (**C**) bones. (**D**,**E**) In tibial cortex, smooth halo-like labeling was observed around osteocytes (arrow) in young bone (**D**), while fluorescent labeling was punctuated in old bone (**E**). Magnified images are shown at the upper right corners of the pictures. PCM labeling intensity was higher in the marrow of young bone (**F**), and while no difference was detected between cortical osteocytes (**G**), the histograms and the percentages of osteocytes expressing low-PCM, medium-PCM, and high-PCM intensity differed in young vs. old bone (**H**).

Different metabolic labeling patterns were found in young and old bones. As expected, robust green labeling was found in areas with high cellular activity, including bone marrow (Figure 3A, red box), primary spongiosa, periosteum, and endosteum, while the tibial cortex (yellow box) showed much weaker labeling intensity (Figure 3A). Marrow showed stronger green labels in the young bone (~40% higher in the averaged intensity of 10 sections per bone) than those in the old bone (Figure 3B,C,F). Fluorescent labeling around osteocytes appeared as a solid halo in the young bone (Figure 3D) but was more punctuated in the old bone (Figure 3E). Although no difference was observed in the average intensity values for osteocytes from young or old mice (Figure 3G), different patterns of the osteocytic PCM intensity were found when the histograms of cell populations were examined: Relative to osteocytes in the old bone, those in the young bone showed a broader distribution with a higher percentage of osteocytes expressing low (15% vs. 8%) and high-intensity (17% vs. 12%; Figure 3H). Labeling intensity was generally weaker around osteocyte dendrites and, thus, was not quantified herein.

### 3.3. Different Labeling Patterns in Young vs. Old Bone

**Figure 4:**
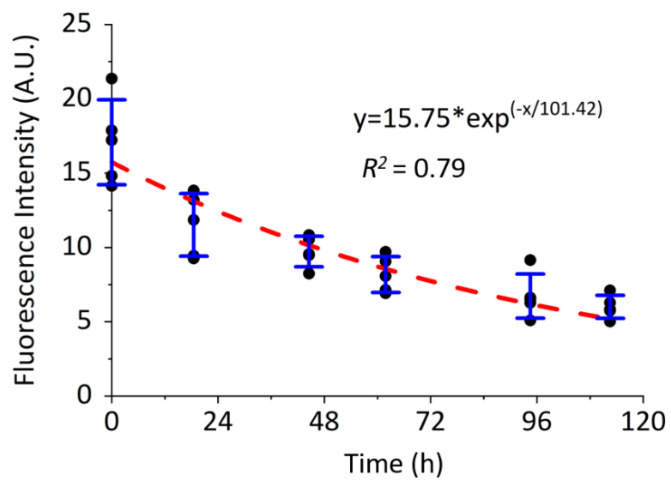
Quantification of in vitro metabolically labelled PCM in pulse-chase experiments. PCM intensity of the MLO-Y4 cells declined exponentially with a half-life of 70 h. N = 5 experiments.

After pulse labeling of the de novo PCM (*t* = 0), the intensity of labeled PCM around the MLO-Y4 cells declined exponentially (y = 15.75*exp^(−x/101.42)^, *R*^2^ = 0.79, Figure 4). The half-life of the osteocytic PCM was estimated to be ~70 h in monolayers of MLO-Y4 cells.

**Figure 5:**
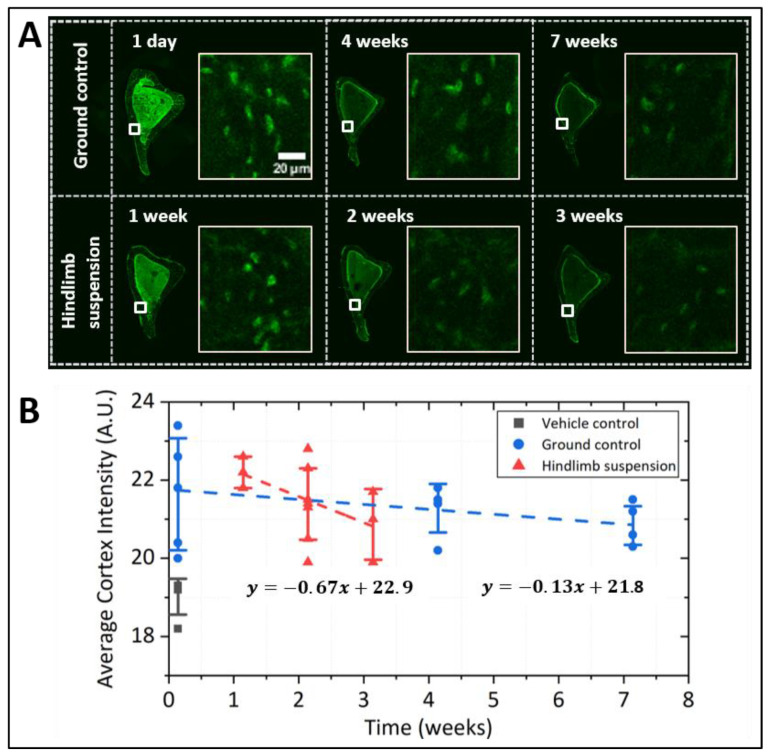
Pulse-chase experiment on metabolically labeled osteocytic PCM in hindlimb suspended mice. (**A**) Representative images of tibial cross sections (left) and magnified regions of the anterior-medial portion of the cortex (white boxes, right) from ground control or hindlimb suspended mice at various time points. (**B**) PCM labeling intensity (averaged across entire sections) showed a linear decrease over time, and the slope was ~5 times steeper in the hindlimb suspended group than the ground control (*p* = 0.13). The decay of PCM labeling intensities, after subtraction of background (the mean value from the vehicle control group), yielded the half-lives of osteocytic PCM at 75 d and 20 d for ground control and hindlimb suspension groups, respectively. Data were measured on sections from both tibiae per mouse, one mouse at the one-day or one-week time point and two mice per time point afterward.

**Figure 6:**
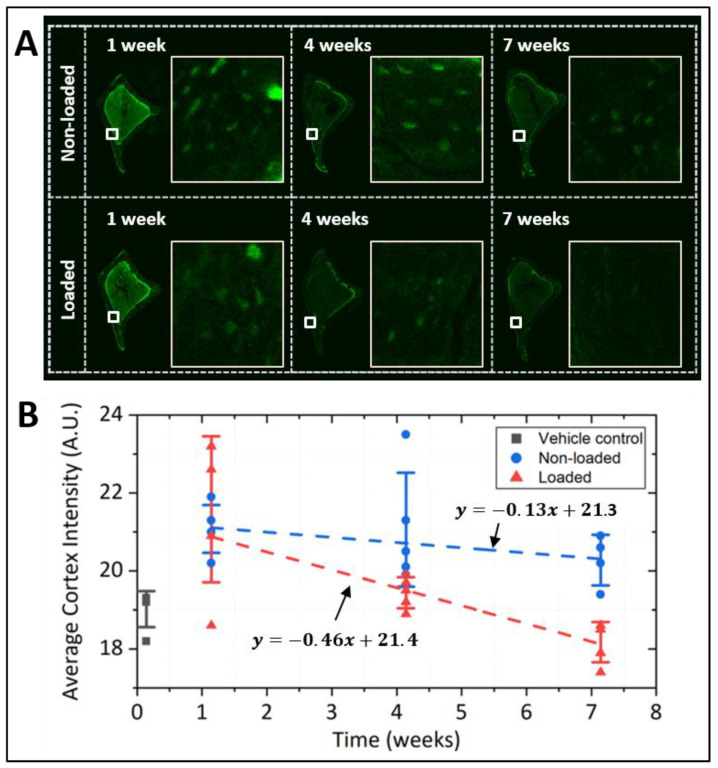
Pulse-chase experiment on metabolically labeled osteocytic PCM in mechanically loaded mice. (**A**) Representative images of tibial cross sections (left) and magnified regions of the anterior-medial portion of the cortex (white boxes, right) from non-loaded and loaded bones at various time points. (**B**) PCM labeling intensity (averaged over the entire cross-section, four sections per time point) showed a linear decrease with time, and the slope was ~3.5 times steeper in the loaded bones than non-loaded bones (*p* = 0.01). The PCM labeling intensities, after subtraction of background (the mean value from the vehicle control group), yielded half-lives of 62 d and 18 d for non-loaded and loaded bones, respectively.

Osteocytic PCM intensity in living bone decreased at a much slower rate than in osteocytic cells in culture. The temporal decay of PCM labeling intensity over 3–7 weeks was shown in images of the tibial cortex (Figure 5 and Figure 6). The ground control group experienced a nearly linear decay of PCM intensity over a period of 7 weeks (y = 21.8 − 0.13x, where x is in the unit of weeks, *R*^2^ = 0.13, Figure 5). PCM turnover was accelerated by hindlimb suspension (Figure 5) and tibial loading (Figure 6). Hindlimb suspension increased the degradation rate as the slope from linear regression became 5-times steeper (y = 22.9 − 0.67x, *R*^2^ = 0.25, Figure 5), although the difference of the two slopes did not reach statistical significance (*p* = 0.13). The half-life of the labeled PCM was estimated to be 75 d and 20 d for ground control and hindlimb suspension groups, respectively. In unilateral tibial loaded mice, non-loaded tibiae showed a slow degradation of osteocytic PCM (y = 21.3 − 0.13x, *R*^2^ = 0.10) with a half-life of 62 d (Figure 6). In contrast, the loaded tibiae showed 3.5-times faster PCM degradation (y = 21.4 − 0.46x, *R*^2^ = 0.64, Figure 6) with a half-life of 18 d. Analysis of covariance showed that degradation rates in the loaded and non-loaded tibiae were significantly different (*p* = 0.01). Vehicle controls showed some background intensity due to non-specific staining of DBCO, which was subtracted in half-life calculations (Figure 5B and Figure 6B).

### 3.4. Mechanical Loading Increased Transcript and Protein Levels of MMP 14 in Bone

**Figure 7:**
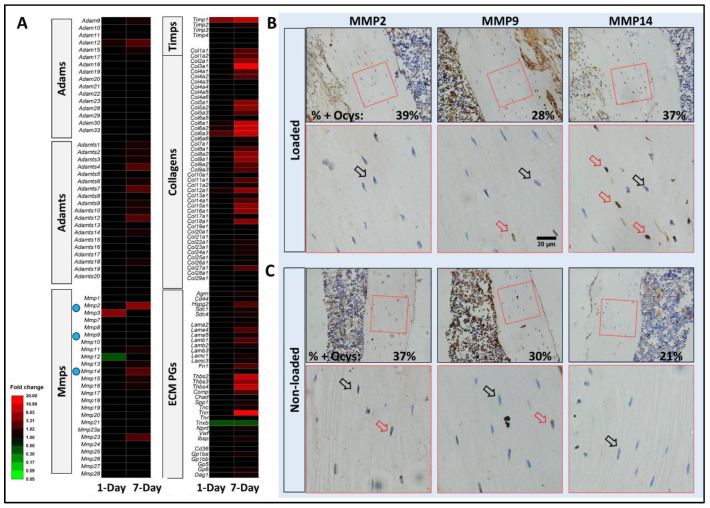
Mechanical loading increased transcripts encoding both matrix proteins and matrix degradation enzymes and MMP14 staining in bone. (**A**) A heat map of fold changes of selected transcripts encoding MMPs, ADAMTS, and ADAMs; as ECM proteoglycans, collagens, and TIMPs, most of which were increased by 7-day mechanical loading as indicated by the overall red increase. (**B**,**C**) Loading increased the expression of MMP14, as the percentage of osteocytes positively labeled with MMP14 (red arrows in magnified regions) over total osteocytes (black arrows on negatively labeled osteocytes) increased in loaded tibiae (37%) compared with non-loaded tibiae (21%). Mechanical loading did not alter IHC staining of MMP2 and MMP9.

Analysis of a previous RNA-sequencing dataset [20] was performed in light of our current hypothesis on the effect of mechanical load on PCM remodeling. Mechanical loading increased levels of transcripts encoding various collagens, heparan sulfate proteoglycans (*Hspg2, Sdc1,* and *Sdc4*), and tissue inhibitor of metalloproteinases (*Timp1*), and more transcripts were elevated after 7-day repetitive loading (Figure 7A). Transcripts encoding matrix degradation enzymes such as MMPs, ADAMTSs, and ADAMs also were increased by loading, especially by 7-day repetitive loading (Figure 7A). For example, 7-day loading resulted in increased transcripts of *Mmp2* (4.6 fold), *Mmp14* (2.3 fold), *Mmp23* (2.7 fold), *Adam12* (2.6 fold), *Adamts4* (2.6 fold), *Adamts7* (2.0 fold), and *Adamts12* (3.0 fold). Further analysis using IHC staining of MMP2, MMP9, and MMP14 showed that MMP14 positive osteocytes were 1.7 times greater in the loaded tibia (37% of all cells) compared to non-loaded tibia (21%, Figure 7B). No significant difference was observed for MMP2 and MMP9 positively labeled osteocytes.

## 4. Discussion

A new semi-quantitative imaging tool was developed and tested to label and quantify osteocytic PCM. Its applications could improve our fundamental understanding of bone mechanobiology, particularly how bone senses, responds, and adapts to mechanical signals. Weinbaum et al. (1994) first proposed an osteocytic PCM model consisting of glycosaminoglycans and established that the bone LCS is the anatomical site for load-induced fluid flow, which activates osteocytes as blood flow stimulates endothelial cells [10]. Although supported indirectly by tracer perfusion studies (reviewed in [8]), osteocytic PCM tethers were not visualized until the breakthrough electron microscopy study by You et al. (2004) [6]. We later identified perlecan, a large linear heparan sulfate proteoglycan, in the LCS [7] and found that perlecan is long and strong enough to serve as a mechanosensor [40]. By using confocal imaging and molecular sieving modeling, we further quantified the spacing of PCM tethers in young adult bone (~13 nm) and aged and perlecan deficient bones (17–23 nm) [25]. Reduced osteocytic PCM in perlecan-deficient mice resulted in decreased fluid drag on sensing tethers [25], suppressed intracellular calcium signaling in osteocytes, impaired the activation of mechanotransduction pathways [20], and, thus, diminished load-induced bone formation in vivo [25]. Although variations of osteocytic PCM tethers have been documented between different genotypes (perlecan deficiency vs. wild type) and ages [25], PCM turnover under various mechanical environment was barely investigated due to technical challenges.

By hijacking a cell’s intrinsic mucin-type *O*-glycosylation pathway and taking advantage of bio-orthogonal click chemistry [31], the labeling method reported in this study enabled us to fluorescently label the newly synthesized osteocytic PCM, as its glycan contents include proteoglycans [7]. Our method was validated from both in vitro and in vivo testing. Morphologically, the method revealed a thin (~1 μm thick) and halo-like labeling pattern surrounding the cell membrane, similar to the ring of newly deposited GFP-tagged collagens around osteocytes [41]. Quantitatively, we also observed decreased labeling intensity in the marrow of old bone, which was in agreement with age-related decreases in total protein and proteoglycan synthesis [42,43,44,45] and O-linked oligosaccharide chains [46]. In addition, we observed that fewer osteocytes showed either high or low PCM intensity in the older bone. It is possible that reduced synthesis with aging accounts for fewer osteocytes with high-PCM intensity and senescence or apoptosis of osteocytes in aged bone [47,48] results in an increased number of empty lacunae and, thus, reduced the count of cells with low PCM intensity. The results supported the applicability of metabolic labeling to study PCM turnover in bones.

Pulse-chase experiments revealed two important findings regarding the dynamics of osteocytic PCM. First, a drastic difference in the PCM half-life was observed between living bones (75 d) and cultured cells (70 h). The 20-fold difference in PCM degradation rates could be accounted for by two possible factors. One is the phenotypic shift of osteocytes from being terminally differentiated living in a natural 3D environment to becoming proliferating in a 2D culture; the other is the more rapid transport of shed PCM components to the surrounding open medium in 2D cultures than the restricted transport through the LCS in living bone tissues. Our previous studies demonstrate that solute transport in LCS is reduced from free diffusion [49], with a greater reduction for larger and more linear molecules [50]. At first glance, the long half-life of in vivo PCM tethers supports the simplified view of them being relatively stable [15,25,40]. However, upon exposure to mechanical loading at moderate-to-high magnitude (5.1 N, 680 με) [51] or the removal of weight bearing, osteocytic PCM undergoes accelerated degradation 3–5 times faster than normal ground control. This suggests that PCM tethers are likely a pool of biomolecules with their steady state being maintained dynamically by a mechanically driven autoregulation process, a concept proposed by Rubin et al. (1999) [52].

The balance of synthesis and degradation processes determines the structure and property of the osteocytic PCM and, hence, the function of osteocytes. It should be noted that our quantitative measures reflect the degradation rates of pre-labeled PCM before the application of tibial loading and disuse, which could affect new PCM synthesis during the loading or disuse period. Considering the literature data regarding protein/matrix synthesis, we anticipated a net decrease in PCM tethers in terms of disuse because of accelerated degradation [52,53] and reduced synthesis [54]. Sparser PCM is associated with lower sieving and increased molecular transport in LCS [25], which could be a compensatory coping mechanism for osteocytes in the absence of load. In the case of mechanical loading, its anabolic effects on matrix synthesis [13,17] could counter the accelerated PCM degradation and result in no change or even denser PCM tethers. The detailed profiles of PCM remodeling would depend on parameters such as magnitude, frequency, and duration of mechanical loading, bone site, and animal age, which can be quantified further in future experiments.

Given the well-accepted anabolic effects of loading [13,17], accelerated PCM degradation in the loaded bone was somewhat surprising. Due to the fact that mechanical loading drives solutes in and out of the LCS [8,51], the loss of PCM labeling could be attributed to enhanced transport of degraded PCM fragments. In order to address this concern, we pulse labeled PCM in two mice and subjected their left tibiae to a single bout of loading for a total of 105 min so that the number of loading cycles was equivalent to the total loading cycles for the entire 7-week experimental duration (5 min/d, 3 d/week). If enhanced transports cause PCM fragments to move out of LCS, PCM intensity would decrease in loaded tibiae compared with the contralateral non-loaded tibiae. However, we did not observe such decreases in PCM intensity by the loading bout. Therefore, the loss of PCM labeling observed in vivo was caused primarily by cell-mediated degradation. We and others [55] detected an increase in MMP14 at transcription and/or protein levels in loaded tibiae. MMP14/MT1-MMP is a membrane-anchored proteinase and an activator of pro-MMP2 and pro-MMP13 [56]. MMP14 can cleave type I, II, and III collagens and proteoglycans [57] and mediate mucin shedding from epithelial cells [58]. Other enzymes such as heparanase also could be involved in PCM degradation, as it is in cartilage [59].

We chose to treat the animals with a modified sugar (Ac_4_GalNAz), because the sugar molecule can be incorporated into cell surface glycans in vivo [32,33] in a timely manner (30–45 min) without any noticeable toxicity [60]. The data presented in this study, thus, were limited to the glycan components of the PCM, although the protein cores in PCM could be studied by using amino acid analogs as the metabolic precursor [31]. Our use of n-acetylgalactosamine analogue allows non-specific labeling of proteoglycans, glycoproteins and glycosphingolipids [37] but cannot identify and quantitate specific contents. In addition, the number of animals used in the study was small. Thus, our findings were an initial test of concept, which means that they need to be confirmed with a larger sample size and more imaging stacks to account for potential spatial variations of PCM expression in young and old bones. We also would like to acknowledge the semi-quantitative nature of measurements using labeling intensity. Although we made an effort to maintain consistency of all experimental parameters including in vivo dosing regimen, sectioning thickness, and imaging settings, labeling intensity data should be treated as being semi-quantitative. In the future, we will improve upon the quantitative aspects of this method by combining quantitative approaches such as mass spectroscopy. 

## 5. Conclusions

This study, for the first time, developed a novel method for visualizing and tracking osteocytic PCM in vitro and in vivo. This method was tested to detect different labeling patterns in young vs. old bones. Application of the method in “pulse-chase” experiments revealed dramatic difference in the “half-life” of PCM of cultured osteocytes (~70 h) and that of osteocytes in vivo (~75 d). When mice were subjected to either 3-week hindlimb unloading or 7-week tibial loading (5.1 N, 4 Hz, 3 d/week), PCM half-life was shortened (~20 d), and degradation accelerated. Matrix metallopeptidase MMP-14 was elevated in mechanically loaded osteocytes, which may contribute to PCM degradation. This study provides a detailed procedure that enables semi-quantitative study of the osteocytic PCM remodeling in vivo and in vitro. We envision that this approach provides a novel and powerful tool for studying osteocytic PCM changes in various physiological and pathological conditions and enhances our understanding of osteocyte function and bone mechanobiology during aging.

## Data Availability

All raw data are available to share upon request.
